# Effects of In Ovo Injection of Coenzyme Q10 on Hatchability, Subsequent Performance, and Immunity of Broiler Chickens

**DOI:** 10.1155/2019/7167525

**Published:** 2019-02-04

**Authors:** Majid Kalantar, Seyed Mahdi Hosseini, Mohammad Reza Hosseini, Mohammad Hassan Kalantar, Farmanullah, Li Guo Yang

**Affiliations:** ^1^Animal Science Department, Qom Agricultural and Natural Source Research and Education Center (AREEO), Qom, Iran; ^2^Key Laboratory of Agricultural Animal Genetics, Breeding and Reproduction, Education Ministry of China, Huazhong Agricultural University, Wuhan, China; ^3^Young Researchers and Elite Club, Saveh Branch, Islamic Azad University, Saveh, Iran; ^4^Department of Animal Science, Islamic Azad University, Saveh Branch, Saveh, Iran; ^5^Research Committee Student, Arak University of Medical Science, Arak, Iran; ^6^Faculty of Veterinary and Animal Sciences, Department of Animal Breeding and Genetics, Lasbela University of Agriculture, Water and Marine Sciences, Uthal, Balochistan, Pakistan

## Abstract

Effects of in ovo injection of Q10 on hatchability, performance (feed intake (FI), body weight gain (BWG), feed/gain ratio (F/G)) traits, and immune status of Ross × Ross 308 broiler chicks, hatched from eggs laid by a 38-week-old breeder flock, were determined through 42 days after hatch. Eggs containing live embryos were injected in the amnion with 0.1 and 0.2 mL Q10 solution on day 18 of incubation. Two controls groups were included as sham and/or as an uninjected group. At 28 and 42 days of age, performance traits, serum enzyme activity, weights of immune organs, and serum antibody titer of viral diseases were determined. Results were shown that hatchability % increased by Q10 on average of 6.54% (P≤0.025) and body weight/egg weight after hatching increased up to 4.74% (P≤0.002), compared with uninjected and sham controls. Injection of Q10 at different levels led to significant increases (P≤0.001) in performance traits all over the rearing period (P<0.05). Weight of immune organs significantly improved compared to uninjected and sham controls (P<0.05). In addition, serum antibody titers of viral diseases as well as serum enzyme activity of AST, ALT, CAT, and SOD were significantly changed by Q10 treated groups than controls (P≤0.01). In conclusion, in ovo injection of Q10 at levels of 0.1 and 0.2 mL led to significant increases in hatchability%, internal egg characteristics, and performance parameters as well as serum enzyme activity, weight of immune organs, and serum antibody titer of ND, AI, and IBD diseases.

## 1. Introduction

The physiological role of coenzyme Q_10_ (CoQ_10_) or ubiquinone is a vitamin-like substance which is the coenzyme for mitochondrial enzymes (complexes I, II, and III) through the inner membrane [1]. Mitochondrial enzymes are essential to oxidize nutrients as a key component of oxidative phosphorylation in the mitochondria and the production of the high-energy phosphate compound (ATP), upon which all cellular functions are facilitated [2, 3]. Besides its bioenergetic function in mitochondrial respiratory chain, CoQ10 is also present in several subcellular fractions or in plasma lipoproteins, where it acts as a powerful lipid-soluble antioxidant [2, 4]. Protective effects of CoQ10 administration were found in experimental models against the deteriorative effect induced by free radicals all over the body cells [3]. Coenzyme Q10, as a potent antioxidant, acts by scavenging reactive oxygen species (ROS) for protecting the embryo against oxidative damage in many degenerative diseases [5].

Development of broiler embryos and hatched chickens are influenced by the yolk nutrient storage. Yolk is the main source of lipids in the egg which supply energy for early development of embryo by oxidative phosphorylation [6]. During initial growth of embryo, rapid oxidative metabolism leads to production of large quantity of free radicals which could be dangerous to embryo [7]. Antioxidants are a critical defense against free radicals, but freshly laid eggs, especially those from birds fed low quality diets, were found to have low concentrations of antioxidants. Thereupon, in ovo injection of antioxidants during incubation may enhance antioxidant qualification of the chicken embryo [8]. Also depression of immunity system due to failure of vaccination, widespread of infectious diseases, and unusual administration of antibiotics lead to impressing immunity responses [9], while malfunction of antioxidant system inside the egg or in the chicken body leads to lower hatchability and subsequent performances [10]. It is well demonstrated that the ratio of esterified short-chain fatty acids is the highest in the tissues of avian embryos on day 18 of incubation, indicating the importance of fatty acid oxidation for energy production in embryos [11]. During the end stage of incubation, especially during the maturate stage, the embryo expends increased amounts of energy [12]. Therefore, the egg coQ10 concentration could be a limiting factor for the *β*-oxidation of fatty acids during emergence from the eggshell. At such times, exogenous supplementation of coQ10 could to be advantageous.

The results of some studies indicated that coQ10 supplementation of hatched chickens diets at different ages resulted in high levels of antibody production for ND, AI, and IBD compared to the negative and positive control groups [13, 14]. However, the degree and quality of response for immune system to in ovo injection of coQ10 or other nutrients depend upon genetics, parent stock age, egg size, and incubation conditions [8].

According to the important function of coQ10 it is essential to use in ovo injection of coQ10 in enhancing the growth and promoting the immune of newly hatched chickens, as well as oxidative prevention of the hatching eggs. Therefore, the aim of this work is to evaluate the effect of in ovo injection of CoQ10 in different dosage into eggs of breeder hens on the hatching performance and posthatch growth as well as serum antioxidant activity and immune response of the young chickens.

## 2. Materials and Methods

### 2.1. Incubation and Injection

The experimental procedure of this study was approved by the Institutional Animal Care and Use Committee of Qom's Agriculture Research Center, Qom, Iran. Ross × Ross 308 broiler hatching eggs from a medium age breeder flock (38 week of age in first stage of production, with average egg weight of 62.5 g, and production percentage was equal to 81%) maintained according to the breeders nutritional schedule were collected (within 3 d of egg collection), weighed, and distributed into 4 groups of 120 eggs each (480 eggs were set totally). Two groups were used as sham control (injected with 0.5 mL commercial diluent) and no injected control. The other 2 groups were injected with 0.1 and 0.2 mL of Q10 solution (1 and 2 mg dissolved in 500 *μ*L of a commercial diluent, respectively) per egg using 25 mm needle based on common methodology [8, 15]. Commercial diluent was included as sham control due to neutralized possible negative responses caused by the stress of injection and handling [8]. On day 18 of incubation, all of the eggs were weighed and then a volume equal to 0.5 mL of treatment solution or commercial diluent was injected into the amnion cavity in depth of 25 mm of each egg using an automated egg injector. Sham-injected control and Q10-injected groups (all of injected solutions containing Q10) were prepared by direct dissolution of Q10 powder (~98% purity; Sigma Chemical Co., St. Louis, MO) in the 100 *μ*L of commercial diluent.

The injection operations were performed under laminar flow system. Under these conditions, temperature of the chamber was maintained at 37°C to avoid thermal shock to the chicken embryos [8, 12]. Before starting the in ovo injection, the point of injection was completely disinfected with 70% ethanol, while the solutions were warmed to 30°C [16]. After injection the injected eggs were returned to the incubator. Any in ovo injection was completed within 20 min out from incubator. Immediately after the injection, the injection site was sealed with sterile paraffin wax and eggs were gently returned to the incubator. On the 19th day of incubation, eggs were transferred to the hatchery and were placed in the certain hatching boxes. Finally, on the day of hatching, chicks were weighed and hatching percentage was recorded.

### 2.2. Bird Management and Feeding

One-day-old hatched chicks were distributed into 4 treatment groups with 4 replicates according to number of hatched chicks per each group. All chicks were reared for six weeks under similar recommended managerial conditions according to manual of Ross 308 broiler management [17]. At the first day, the lighting schedule was 23h light and 1 h darkness at 32°C. It was subsequently reduced 3°C each week until the end of the third week and then after that was constant at 22°C. Chickens were fed a basal diet based on NRC [18], to meet the standard nutrient requirements of poultry (Table 1). Diets in mash form and fresh water were offered to chickens ad libitum. Weight gain (WG) and feed intake (FI) were measured weekly and feed conversion ratio (FCR) was calculated accordingly.

### 2.3. Serum Enzyme Activity

At day one and the end of trail (42 days of age), 10 birds per treatment were selected for blood sampling (2-3mL/bird). Blood samples of day old chickens were taken directly from the heart and for 42-day-old chickens were taken from brachial vein to measure serum concentration of hepatic enzymes of AST, ALT, CAT, and SOD [8, 15]. Blood samples were transferred to tubes and kept at room temperature to clot. Blood samples were then centrifuged at 2,500 × g for 10 min at 4°C to separate the impurity of the samples. The separated serum samples were measured using commercial kits (Sigma-Aldrich Co., St. Louis, MO, USA) and a spectrophotometer according to a colorimetric method [19].

### 2.4. Immunological Assay

Chickens were vaccinated with Newcastle Disease (ND), Avian Influenza (AI), and Infectious Bronchitis (IB) at age of 21 and 35 days according to local veterinarian recommendations schedule in order to challenge the immunity system of chickens. Four birds from each replicate were randomly selected and blood samples were taken from wing vein on 28 and 42 days of age (7 days after challenging by vaccine). Serum was separated by centrifugation of coagulated blood at 3000 r/min for 15 min. Antibodies titration against ND, AI, and IB diseases was performed using ELISA by specific kits of OVATEC® Plus, SERELISA® Rabies (Synbiotics, USA), and BIA-CK® 121 (Biochek, Netherlands), respectively.

### 2.5. Ethical Consideration

As previously described, the experimental procedure of this study was approved by the Institutional Animal Care and Use Committee of Qom's Agriculture Research Center. Meanwhile, all the methods used for experimental birds in this study were ethically approved by Ethical Review Committee of Public Health College and Medical Sciences of Qom University [20], Qom, Iran. Detailed experimentation procedures in birds as described in this manual for animal handling and treatment guidelines were carefully followed.

### 2.6. Measurements

On the day of hatch, hatchability percentage, egg weight, and body weight of chickens were measured. At this time two newly hatched chicks from each replicate were randomly selected and the weight of internal organs such as bursa of Fabricius, spleen, and yolk sac was recorded. During the experiment performance parameters including daily feed intake and weekly gain were recorded and for 1 to 21 and 22 to 42 days as well as all over the experiment period (1 to 42 days) FI, WG, and FCR were calculated. Also on day 42 after hatch two birds from each replicate were randomly selected and slaughtered and weight of bursa of Fabricius, spleen, and liver was determined. On days 28 and 42 after hatch, in order to determine the serum enzymes and immunological parameters, blood samples from two randomly selected birds were collected. After overnight clotting of blood samples at 4°C, the samples were centrifuged at 1,000 ×g for 20 min and sent to laboratory for further measurements [8, 15, 19].

### 2.7. Statistical Analysis

At the end measured data were analyzed for normal distribution using the NORMAL option of the Univariate procedure of SAS software (SAS, 2008). Pen was considered as the experimental unit and data were analyzed based on completely randomized design by the GLM procedure. Statistical differences between the means were established using a Duncan's Multiple Range Test (DMRT) at the level of P≤0.05.

## 3. Results

According to the results of Table 2, hatchability percentage was significantly increased by in ovo injection of 0.1 and 0.2 mL of Q10 per egg rather uninjected and sham controls (P≤0.025). Moreover, significant differences were found in body weight of chickens (P≤0.002). In ovo injection of Q10 (0.1 and 0.2 mL) led to significant increases in body weight/egg weight after hatching (P≤0.001), while the egg weight between the treatments was approximately fixed.

Table 3 shows that in ovo injection of Q10 at levels of 0.1 and 0.2 mL per egg led to significant increases in feed intake and body weight gain in both 1-21 and 22-42 days of age (P<0.05). Significant differences were found in performance parameters in whole experimental period (1-42 days) (P<0.05).

According to the results presented in Table 4, significant differences were found in weight of bursa of Fabricius (P<0.014) and yolk sac (P<0.001) at day of hatching but weight of spleen was not significantly different between treatments. But at 42 days of age CoQ10 at levels of 0.1 and 0.2 mL per egg led to significant increases (P<0.05) in weight of all immune organs including bursa of Fabricius, spleen, and liver.

Table 5 shows that in ovo injection of Q10 at levels of 0.1 and 0.2 mL per egg led to significant increases in serum enzyme activity in both 1-21 and 22-42 days of age (P≤0.01). Significant differences were found in activity of AST, ALT, and antioxidant enzymes in the serum of chickens (P≤0.01).

In ovo injection of Q10 at levels of 0.1 and 0.2 mL per egg led to significant differences in ND, AI, and IBD antibody titer at 28 days of age (P<0.05). Moreover, significant increases were found in ND, AI, and IBD antibody titer as compared to uninjected and sham groups at 42 days of age (P<0.05) (Table 6).

## 4. Discussion

In ovo injection has become an important tool to administer vaccines and essential nutrients into egg in the hatcheries [6]. Nevertheless, some basic precautions including proper disinfection of the hatching eggs must be taken into account in order to achieve the best results with this procedure [21, 22].

It has been demonstrated that an effective way to supply embryos with essential nutrients could be in ovo injection of nutrients [21, 23]. In this regard in ovo injection of antioxidants could improve embryo development and can prevent oxidative damage [8]. Previous studies have shown that the absorption of exogenously administering CoQ10 due to its lipophilic nature and relatively high molecular weight is slow and limited [24, 25]. However, increasing its solubility in aqueous medium should increase the bioavailability [26, 27]. So developing a soluble form of CoQ10 exhibits increased water solubility [26] and better bioavailability in comparison to powder and oil-based CoQ10 forms [27, 28].

Results of this experiment show that CoQ10 increased hatchability percentage and posthatch performance parameters, immune organ weights, activity of antioxidant serum enzymes, and serum antibody titer of common viral diseases such as ND, AI, and IBD. Hatchability percentage and body weight of newly hatched chickens were improved by in ovo injection of CoQ10 at levels of 0.1 and 0.2 mL of Q10 per egg. The result of hatchability percentage is consistent with similar studies (Coškun et al., 2014) [22], so the hatchability percentage was influenced by the type of injected substance and site of injection into the eggs as well as proper disinfection process [8]. Exogenous supplementation of CoQ10 at critical time of fatty acid oxidation can be useful in reducing the production of free radicals that cause a serious damage to the embryo cellular membranes [3–5] and increase lipid utilization for energy production to improve hatchability [5, 29–31]. Therefore, it is concluded that increased hatchability percentage in this experiment after in ovo injection of CoQ10 may be due to the improvement of the antioxidant status of the eggs or protection effects of the CoQ10 against oxidation (Table 5). It is reported that higher body weight was also obtained by extra supplementation of CoQ10 due to the prevention of hydroperoxides of fatty acids and more energy uptake by CoQ10 which enhance the embryonic growth [22, 30, 31].

Results also showed that in ovo injection of Q10 at levels of 0.1 and 0.2 mL of Q10 per egg led to significant increase in performance parameters such as feed intake, body weight gain, and feed conversion rate in both 1-21 and 22-42 days of age of broilers as well as in the entire experimental period. Coordinated by the effect of in ovo injection of Q10 on hatchability and body weight of hatched chickens, it is assumed that in ovo injection of Q10 had no adverse effect on the growth of the developing embryo and posthatch growth of broilers. At 21 days of age, 0.1 and 0.2 mL of Q10 per egg groups had on average 5.63 and 6.84 g higher weight gain compared to uninjected and/or sham control groups, respectively. At 42 days of age, weight gain for 0.1 and 0.2 mL of Q10 per egg was on average 5.63 and 7.73 g higher than uninjected and sham control groups, respectively. On average, FCR during first 21 days of growth period was 0.11 and in second 21 days of growth period was 0.08 lower than uninjected and sham control groups, respectively. Significant differences which were found in FI, BWG, and FCR by treatments are in line with previous study with in ovo injection of Q10 [22]. Reports demonstrated that the degree of response to in ovo injection depend on genetic, parent stock age, egg size, disinfection, and incubation conditions [6, 8].

Previous studies supported this assumption and showed that coQ10 supplementation or exogenous Q10 injected into egg at the time of incubation is affecting posthatch growth [22, 30–33]. Krizman et al. [34] suggested that continuous supplementation (1 to 42 days) of CoQ10 improved the body weight over the control group. Higher body weight of chickens in this study may be due to effect of coQ10 on energy efficiency during the metabolism of nutrients at the time of incubation or posthatch growing period as well as its antioxidative properties. Fathi [35] reported that both 20 and 40 mg per kg CoQ10 supplementation improved feed conversion ratio at whole period of study (3-6 weeks) in treated birds compared to control group. CoQ10 exerts a fundamental role in bioenergetics process in the cells as a cofactor in the respiratory chain to electron transport chain and is therefore essential for the production of ATP, which support the growth all over the rearing period especially in newly hatched chickens [35, 36].

The weight of immune organs and yolk sac in coQ10 treated groups was higher than their counterpart at day of hatching. Also the weight of bursa of Fabricius, spleen, and liver for mentioned groups was higher at 42 days of age. Similar observations was reported by Nemati et al. [13] who suggested that coQ10 singly or in combination with vitamin E can increase the weight of immune organs including bursa of Fabricius and spleen especially under cold stress condition. The explanation of these observations is difficult because of limited study in this regard, but improvement in immunity after administering or in ovo injection of CoQ10, vitamin E, or other nutrients was seen in number of experiments and was expected due to preventive effect of coQ10 against cellular oxidative agents [8, 10, 33]. A hypothesis declares that CoQ10 antioxidant properties result in standoff free radicals and contribute to prevention of lipid peroxidation which is an important step in suppression of immune organs to produce normal immunological products (Yokoyama et al. 1996); [35].

An experiment result has shown that both options may be involved by supplementation of CoQ10, which, at first, increased coQ10 production in liver tissue and, in the second stage, reduced the oxidative damage which may additionally save the endogen CoQ10 content. Observations on the measurement of serum enzyme activity of selected antioxidant enzymes in this experiment are consistent with the recent hypothesis (Table 5). This experiment has also shown that the increase in CoQ10 plasma concentrations in young chickens is greater than in adult hens during the supplementation with CoQ10 [37].

The results of this study indicated that the antibody titer of ND disease was significantly increased during the entire of rearing period by direct injection of coQ10 into eggs. Moreover, significant improvement was observed in antibody titers of AI and IBD disease at both 28 and 42 days of age. Though it seems that the difference in hatchability percentage has been observed between the two control groups (the sham and uninjected groups) related to the effect of injection on egg fertility and hatching performance. Higher improvements in immunity system with regard to higher antibody production by in ovo injection of coQ10 in present study are in accordance with the results of many studies [13, 14, 31]. It is due to special effect of antioxidant components that protects tissue of immunological organs against destruction [38], also influences of coQ10 on helper T-cells, phagocytosis activity, and prostaglandin synthesis in lymphoid organelles [8, 13]. The antioxidant status of hatched chick's tissues enhances by coQ10 which protects lipid membranes from radical oxygen species [3–5]. In addition, coQ10 reduces oxidative damage and increases proliferation of B-cells and thereby leads to higher immunological productions and better antibodies responses [10, 13, 33].

The results of this study are in agreement with Nemati et al. [13] and Asadi et al. [14], who reported that coQ10 supplementation of hatched chickens diets at different ages were high responders for antibody production of ND, AI, and IBD compared to the negative and positive control groups. However, the degree of response for immune system (humoral and cell mediated immune systems) to in ovo injection depends upon genetics, parent stock age, egg size, and incubation conditions [8]. The growth of the neonatal birds is dependent on residual nutrients found in the yolk sacs that have been discharged during the hatching process [6, 21].

## 5. Conclusion

According to the results of this study, in ovo injection of Q10 into the fertile eggs of broiler breeders could lead to significant increase in hatchability%, internal egg characteristics, and performance parameters as well as serum enzyme activity, weight of immune organs, and serum antibody titer of viral diseases. Recommended levels based on the results of this experiment are 0.1 and 0.2 mL of Q10 solution per egg on day 18 of incubation. Due to positive effects of Q10 to prevent oxidative damage to the embryo it seems that this method could be regarded as a possible method to improve mentioned parameters in newly hatched chickens or even for older chickens.

## Figures and Tables

**Table 1:** Basal diet composition at 1 to 42 days of age (% or as stated).

Item	Starter	Grower
	(1 to 21 days of age)	(22 to 42 days of age)
Corn grain	53.5	53.8
Soybean meal	41.3	38.7
Soybean oil	1	3
Calcium carbonate	1.65	1.63
Dicalcium phosphate	1.75	1.72
Mineral and vitamin premix^1^	0.5	0.5
Common salt	0.3	0.44
Methionine	0.44	0.41
Lysine	1.1	1.1

Total	100	100

Metabolizable energy (kcal/kg)	3050	3000
Crude protein	23	21.54
Calcium	1	0.93
Available phosphorous	0.45	0.45
Calcium: phosphorous	2.22	2.07
Energy: protein	132.61	139.27

^1^Mineral premix: Mn, 80 mg; Zn, 84.5 mg; Fe, 80 mg; Cu, 5 mg; I, 1.0 mg; Co, 0.48 mg; Se, 0.30 mg/kg of diet. Vitamin premix: Vitamin A, 11,000 IU (retinol); Vitamin D3, 3,000 IU; Vitamin E, 50 mg (DL-*α*-tocopheryl acetate); Vitamin K3, 5 mg; tiamin, 2 mg; Riboflavin, 8 mg; calcium pantothenate, 12.40 mg; niacin, 50 mg; pyridoxine, 7 mg; pholic acid, 2mg; Vitamin B12, 1.60 mg; biotin, 5 mg; choline chloride, 1,100 mg; antioxidant, 100 mg/kg of diet.

**Table 2:** Effect of different levels of Q10 on hatchability (%), body weight, and egg weight (g) at hatching time.

Item	Hatchability (%)	Body weight (g)	Egg weight (g)	Body weight/ Egg weight
Sham control	83^c^	40.37^b^	60.14	67.13^b^
Un-injected control	88.5^b^	41.82^ab^	60.54	69.08^ab^
Q10, 0.1 mL/egg	92^a^	42.85^a^	60.78	70.50^a^
Q10, 0.2 mL/egg	91.5^a^	43.43^a^	60.07	72.30^a^
SEM	1.27	0.54	0.28	2.26
P-value	0.025	≤0.002	0.824	≤0.001

Means with common letters in the same columns are not significantly different (P>0.05). SEM: standard error of the means; FI: feed intake; WG: weight gain; FCR: feed conversion ratio; Q10: coenzyme Q10.

**Table 3:** Effect of different levels of Q10 on feed intake, body weight gain, and FCR of chickens at different ages (g).

Item	Sham control	Un-injected control	Q10, 0.1 mL/egg	Q10, 0.2 mL/egg	SEM	P-value
		1-21 days of age				

FI (g)	46.22^b^	46.83^b^	51.57^a^	53.42^a^	1.16	0.009
WG (g)	26.91^b^	27.02^b^	32.59^a^	33.80^a^	1.13	0.044
FCR	1.72^a^	1.73^a^	1.60^b^	1.58^b^	0.04	

		22-42 days of age				

FI (g)	151.46^b^	149.60^b^	152.17^ab^	152.38^a^	1.44	0.034
WG (g)	75.34^ab^	74.17^b^	79.62^a^	78.45^a^	1.39	0.003
FCR	2.01^a^	2.02^a^	1.91^b^	1.94^b^	0.03	0.001

		1-42 days of age				

FI (g)	104.85^b^	106.^b^	111.58^a^	111.54^a^	1.6	0.002
WG (g)	52.75^b^	52.05^b^	58.03^a^	60.13^a^	1.24	0.047
FCR	1.99^a^	2.06^a^	1.93^b^	1.86^b^	0.04	0.039

Means with common letters in the same columns are not significantly different (P>0.05). SEM: standard error of the means; FI: feed intake; WG: weight gain; FCR: feed conversion ratio; Q10: coenzyme Q10.

**Table 4:** Effect of different levels of Q10 on immune organ and yolk sac weights (g) of chicken at different ages.

Item	Sham control	Un-injected control	Q10, 0.1 mL/egg	Q10, 0.2 mL/egg	SEM	P-value
		at day of hatching (g/100g BW)				

Bursa of Fabricius	0.193^b^	0.189^b^	0.207^a^	0.207^a^	0.020	0.014
Spleen	0.071	0.073	0.071	0.078	0.021	0.537
Yolk sac	8.4^a^	7.82^a^	7.27^b^	7.04^b^	0.111	0.001

		At 42 days of age (g/100g BW)				

Bursa of Fabricius	0.082^b^	0.075^b^	0.111^a^	0.112^a^	0.01	0.002
Spleen	0.093^b^	0.103^b^	0.145^a^	0.141^a^	0.01	0.012
Liver	3.49^a^	3.45^a^	2.97^b^	2.51^c^	0.06	0.011

Means with common letters in the same columns are not significantly different (P>0.05). SEM: standard error of the means; Q10: coenzyme Q10.

**Table 5:** Effect of different levels of Q10 on serum enzyme activity of chicken at different ages.

Item	Sham control	Un-injected control	Q10, 0.1 mL/egg	Q10, 0.2 mL/egg	SEM	P-value
		at day of hatching (U/mL)				

AST	219^b^	208^b^	187^a^	186^a^	10.2	0.010
ALT	3.33	3.23	2.33	2.30	0.21	0.001
CAT	6.24^b^	6.18^b^	8.27^a^	8.64^a^	0.11	0.001
SOD	149.8^b^	150.6^b^	172.8^a^	177.6^a^	5.33	0.001

		At 42 days of age (U/mL)				

AST	198^a^	201^a^	166^b^	162^b^	8.21	0.002
ALT	3.11^a^	3.27^a^	2.67^b^	2.45^b^	0.13	0.012
CAT	4.12^b^	5.08^b^	7.82^a^	8.16^a^	0.17	0.001
SOD	139.8^b^	141.6^b^	168.9^a^	171.2^a^	4.95	0.001

Means with common letters in the same columns are not significantly different (P>0.05). SEM: standard error of the means; AST: aspartate amino transferase; ALT: alanine amino transferase; CAT: catalase; SOD: superoxide dismutase; Q10: coenzyme Q10.

**Table 6:** Effect of different levels of Q10 on serum antibody titer of chicken at different ages (log_2_).

Item	Sham control	Un-injected control	Q10, 0.1 mL/egg	Q10, 0.2 mL/egg	SEM	P-value
		at days of 28				

ND	503.72^b^	526.84^b^	611.65^ab^	643.32^a^	21.63	0.022
AI	334.22^c^	323.67^c^	376.23^b^	411.52^a^	8.63	0.031
IBD	168.61^c^	152.11^c^	178.75^b^	193.54^a^	4.82	0.043

		at days of 42				

ND	1256.63^d^	1435.18^c^	1613.54^b^	1936.23^a^	41.26	0.008
AI	803.23^c^	699.41^d^	913.44^b^	1012.42^a^	31.85	0.033
IBD	542.25^d^	642.75^c^	787.25^b^	963.50^a^	19.44	0.002

Means with common letters in the same columns are not significantly different (P>0.05). SEM: standard error of the means; ND: Newcastle disease; AI: avian influenza; IB: infectious bronchitis; IBD: infectious bursal disease; Q10: coenzyme Q10.

## Data Availability

Data will be available on request

## References

[1] Lenaz G., Fato R., Formiggini G., Genova M. L. (2007). The role of Coenzyme Q in mitochondrial electron transport. *Mitochondrion*.

[2] Rauchová H., Drahota Z., Lenaz G. (1995). Function of Coenzyme Q In The Cell: Some Biochemical and Physiological Properties. *Physiological Research*.

[3] Littarru G. P., Tiano L. (2007). Bioenergetic and antioxidant properties of coenzyme Q10: recent developments. *Molecular Biotechnology*.

[4] Bentinger M., Brismar K., Dallner G. (2007). The antioxidant role of coenzyme Q. *Mitochondrion*.

[5] Noh Y. H., Kim K.-Y., Shim M. S. (2013). Inhibition of oxidative stress by coenzyme Q10 increases mitochondrial mass and improves bioenergetic function in optic nerve head astrocytes. *Cell Death & Disease*.

[6] Uni Z., Ferket R. P. (2004). Methods for early nutrition and their potential. *World's Poultry Science Journal*.

[7] Cherian G., Sim J. S. (1997). Egg Yolk Polyunsaturated Fatty Acids and Vitamin E Content Alters the Tocopherol Status of Hatched Chicks. *Poultry Science*.

[8] Salary J., Sahebi-Ala F., Kalantar M., Matin H. R. H. (2014). In ovo injection of vitamin E on post-hatch immunological parameters and broiler chicken performance. *Asian Pacific Journal of Tropical Biomedicine*.

[9] Chen H., Li D., Chang B., Gong L., Dai J., Yi G. (2003). Effects of Chinese herbal polysaccharides on the immunity and growth performance of young broilers. *Poultry Science*.

[10] Niu Z. Y., Liu F. Z., Yan Q. L., Li W. C. (2009). Effects of different levels of vitamin E on growth performance and immune responses of broilers under heat stress. *Poultry Science*.

[11] Rinaudo M. T., Curto M., Bruno R., Piccinini M., Marino C. (1991). Acid soluble, short chain esterified and free carnitine in the liver, heart, muscle and brain of pre and post hatched chicks. *International Journal of Biochemistry*.

[12] Keralapurath M. M., Keirs R. W., Corzo A., Bennett L. W., Pulikanti R., Peebles E. D. (2010). Effects of in ovo injection of L-carnitine on subsequent broiler chick tissue nutrient profiles. *Poultry Science*.

[13] Nemati M. H., Shahir M. H., Harakinezhad M. T., Lotfallahyan H. (2015). Humeral and Cellular Immunity Response of Broiler Under Cold Stress Condition to Vitamin E and Coenzyme Q10. *Research on Animal Production*.

[14] Asadi H., Sadeghi A. A., Eila N., Aminafshar M. (2016). Carcass traits and immune response of broiler chickens fed dietary L-carnitine, coenzyme Q10 and ractopamine. *Revista Brasileira de Ciência Avícola*.

[15] Bhattacharyya A., Majumdar S., Bhanja S. K., Mandal A. B., Kadam M. (2017). Effect of maternal dietary manipulation and in ovo injection of nutrients on the hatchability indices, post-hatch growth, feed consumption, feed conversion ratio and immunocompetence traits of turkey poults. *Journal of Applied Animal Research*.

[16] Coskun I., Akkan A., Erener G. (2018). Effects of in ovo injection of lysine and methionine into fertile broiler (parent stock) eggs on hatchability, growth performance, caecum microbiota, and ileum histomorphology. *Revista Brasileira de Zootecnia*.

[17] ROSS Management Handbook (2009). *Ross broiler management handbook*.

[18] National Research Council (NRC) (1994). *Nutrient requirements of poultry*.

[19] Antolovich M., Prenzler P. D., Patsalides E., McDonald S., Robards K. (2002). Methods for testing antioxidant activity. *Analyst*.

[20] Ethical Guidelines. ERCPHC

[21] Uni Z., Ferket P. R., Tako E., Kedar O. (2005). In ovo feeding improves energy status of late-term chicken embryos. *Poultry Science*.

[22] Eslami M., Salarmoini M., Tasharrofi S. (2014). Effects of In-ovo Injection of Different Nutrients on the Hatchability and Hatchability and growth Performance in Broilers. *Journal of Livestock Science and Technologies*.

[23] Bello A., Zhai W., Gerard P. D., Peebles E. D. (2013). Effects of the commercial in ovo injection of 25-hydroxycholecalciferol on the hatchability and hatching chick quality of broilers. *Poultry Science*.

[24] Hsu C., Cui Z., Mumper R. J., Jay M. (2003). Preparation and characterization of novel coenzyme Q10 nanoparticles engineered from microemulsion precursors. *AAPS PharmSciTech*.

[25] Bhagavan H. N., Chopra R. K. (2006). Coenzyme Q10: Absorption, tissue uptake, metabolism and pharmacokinetics. *Free Radical Research*.

[26] Prošek M., Šmidovnik A., Fir M. Water soluble form of coenzyme Q10 in a form of an inclusion complex with beta-cyclodextrine, process of preparing, and use thefore.

[27] Prosek M., Butinar J., Lukanc B. (2008). Bioavailability of water-soluble CoQ10 in beagle dogs. *Journal of Pharmaceutical and Biomedical Analysis*.

[28] Žmitek J., Šmidovnik A., Fir M. (2008). Relative bioavailability of two forms of a novel water-soluble coenzyme Q10. *Annals of Nutrition and Metabolism*.

[29] Honda K., Kamisoyama H., Motoori T., Saneyasu T., Hasegawa S. (2010). Effect Of dietary coenzyme Q10 on cholesterol metabolism in growing chickens. *Journal of Poultry Science*.

[30] Gopi M., Purushothaman M. R., Chandrasekaran D. (2014). Effect of dietary coenzyme Q10 supplementation on the growth rate, carcass characters and cost effectiveness of broiler fed with three energy levels. *SpringerPlus*.

[31] Gopi M., Purushothaman M. R., Chandrasekaran D. (2015). Influence of coenzyme Q10 supplementation in high energy broiler diets on production performance, hematological and slaughter parameters under higher environmental temperature. *Asian Journal of Animal and Veterinary Advances*.

[32] Geng A. L., Guo Y. M., Yang Y. (2004). Reduction of ascites mortality in broilers by coenzyme Q10. *Poultry Science*.

[33] Geng A., Li B., Guo Y. (2007). Effects of dietary L-carnitine and coenzyme Q10 at different supplemental ages on growth performance and some immune response in ascites-susceptible broilers. *Archives of Animal Nutrition*.

[34] Krizman P. J., Prosek M., Smidovnik A., Eissa A. H. A. (2012). Poultry products with increased content of CoQ10 prepared from chickens fed with supplemental CoQ10. *Trends in Vital Food and control engineering*.

[35] Fathi M. (2015). Effects of Coenzyme Q10 Supplementation on Growth Performance, some Hematological Parameters, Plasma Enzymes Activities in Broilers with Pulmonary Hypertension Syndrome (PHS. *Iranian Journal of Applied Animal Science*.

[36] Bhagavan H. N., Chopra R. K. (2009). Coenzyme Q10: Absorption, tissue uptake, metabolism and pharmacokinetics. *Free Radical Research*.

[37] Krizman P. J., Smidovnik A., Wondra A. G., Krizman M., Prosek M. (2013). Effects of dietary CoQ_10_ and *α*-lipoic acid on CoQ_10_ levels in plasma and tissues of eggs laying hens. *Journal of biomedical science and engineering*.

[38] Shivappa Nayaka H. B., Umakantha B., Wilfred Ruban S., Murthy H. N. N., Narayanaswamy H. D. (2012). Effect of neem, turmeric, vitamin e and their combinations on immune response in broilers. *Global Veterinaria*.

